# Young asteroidal fluid activity revealed by absolute age from apatite in carbonaceous chondrite

**DOI:** 10.1038/ncomms12844

**Published:** 2016-09-29

**Authors:** Ai-Cheng Zhang, Qiu-Li Li, Hisayoshi Yurimoto, Naoya Sakamoto, Xian-Hua Li, Sen Hu, Yang-Ting Lin, Ru-Cheng Wang

**Affiliations:** 1 State Key Laboratory for Mineral Deposits Research, School of Earth Sciences and Engineering, Nanjing University, Nanjing 210046, China; 2 Department of Natural History Sciences, Hokkaido University, Sapporo 060-0810, Japan; 3 State Key Laboratory of Lithospheric Evolution, Institute of Geology and Geophysics, Chinese Academy of Sciences, Beijing 100029, China; 4 Isotope Imaging Laboratory, Creative Research Institution Sousei, Hokkaido University, Sapporo 001-0021, Japan; 5 Key Laboratory of Earth and Planetary Physics, Institute of Geology and Geophysics, Chinese Academy of Sciences, Beijing 100029, China

## Abstract

Chondritic meteorites, consisting of the materials that have formed in the early solar system (ESS), have been affected by late thermal events and fluid activity to various degrees. Determining the timing of fluid activity in ESS is of fundamental importance for understanding the nature, formation, evolution and significance of fluid activity in ESS. Previous investigations have determined the relative ages of fluid activity with short-lived isotope systematics. Here we report an absolute ^207^Pb/^206^Pb isochron age (4,450±50 Ma) of apatite from Dar al Gani (DaG) 978, a type ∼3.5, ungrouped carbonaceous chondrite. The petrographic, mineralogical and geochemical features suggest that the apatite in DaG 978 should have formed during metamorphism in the presence of a fluid. Therefore, the apatite age represents an absolute age for fluid activity in an asteroidal setting. An impact event could have provided the heat to activate this young fluid activity in ESS.

## Abstract (web summary)

Chondritic meteorites formed in the early solar system and may tell us about primary processes at that time. Here, Zhang *et al*. report an absolute ^207^Pb/^206^Pb isochron age (4,450±50 Ma) of apatite from a carbonaceous chondrite constraining timing of fluid activity in meteorites.

## Graphical abstract

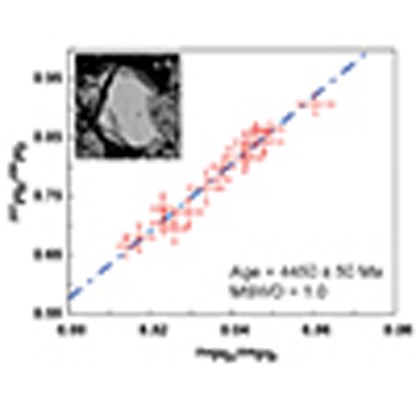

Chondritic meteorites (chondrites) are composed of the solid minerals that have formed in the early solar system (ESS). They are the most precious samples to constrain primary processes that have been prevailed in the solar nebula^1^,^2^. However, most of the chondritic meteorites have been affected by later fluid activity to various extents^3^,^4^,^5^. Fluid activity has changed the mineralogy, chemistry and isotope compositions of chondrites through fluid–mineral interactions (that is, aqueous alteration, metasomatism and fluid-assisted metamorphism^6^). Constraining when and where these fluid–mineral interaction events took place is important to understand the thermal histories of chondrite parent bodies and the temporal-spatial evolution of fluids in the solar nebular and asteroidal settings^7^. The formation time of secondary minerals can further constrain the heating sources that have driven the fluid–mineral interaction events in ESS (for example, decay of ^26^Al, heating during accretion of chondritic components to form asteroidal parent bodies and impact heating). Previous investigations have dated a few secondary minerals (for example, carbonate minerals, ferroan olivine, magnetite, nepheline and grossular) that have formed by low-temperature and metamorphic processes in the presence of fluids, based on short-lived isotopic systematics (for example, ^53^Mn–^53^Cr, ^129^I–^129^Xe and ^26^Al–^26^Mg systematics^7^). Most of these studies revealed that fluid activity in ESS has started within 1–2 Myr after the formation of Allende Ca,Al-rich inclusions (CAIs) and lasted up to 15 Myr (refs 5, 7). A few studies, however, found no resolvable excess of daughter nuclides^8^, indicating an extended period of fluid activity (>25 Myr). However, no absolute ages have been reported for constraining fluid activities in ESS up to date. It also remains unknown how late fluid activities could be present in ESS and whether fluid activity is coupled with young metamorphic processes. This is partly due to the fact that dating using short-lived isotope systems is only applicable to events that occurred several to tens of millions after the solar system formation. In addition, to date, most of the secondary minerals (for example, phyllosilicate, carbonate and magnetite) in chondritic meteorites are not suitable for long-lived isotope geochronology.

Apatite is an important volatile-rich mineral Ca_5_(PO_4_)_3_(F,Cl,OH) in chondrites. Apatite and merrillite [Ca_9_NaMg(PO_4_)_7_] in chondrites are thought to be secondary minerals, due to the large difference in condensation temperatures for Ca and P (refs 9, 10). This is consistent with the absence of Ca–phosphate minerals in most primitive chondrites^1^. The formation of apatite in chondrites is usually attributed to fluid activity^5^,^9^,^11^,^12^, although their formation conditions may vary in different chondrites of various petrologic types (from type 1 to type 6). With the developments of uranium–lead (U–Pb) geochronology with secondary ion mass spectrometer (SIMS), apatite can be dated with detailed petrographic context in polished thin sections^13^,^14^,^15^. Therefore, combined with petrographic and geochemical observations, the age of apatite in chondrites can constrain the absolute ages of fluid activity in ESS and/or subsequent heating events (for example, refs 16, 17).

DaG 978 is an ungrouped and low petrologic type (∼3.5) carbonaceous chondrite with a low abundance of volatile elements^18^. It contains many relatively coarse chlorapatite grains (up to 280 μm in size^19^). Here we report on the petrography, mineralogy, rare earth elements (REEs) and oxygen isotopic compositions, and U–Pb isotopic systematics of the apatite in DaG 978. Our data reveal that the apatite in DaG 978 could have formed during a metamorphic event in the presence of fluid. Its age represents the first absolute age of fluid activity in ESS.

## Results

### General petrography and mineralogy of DaG 978

The detailed petrography and mineralogy of DaG 978 have been reported in ref. 19. The most important features indicative of secondary processes are briefly described here. DaG 978 contains well-defined chondrules, refractory inclusions and fine-grained matrix (mainly, olivine, pyroxene and plagioclase). However, most of the olivine grains in chondrules and refractory inclusions from DaG 978 are Fe rich (Mg#[≡100 × Mg/(Mg+Fe)]=65–76), whereas their associated low-Ca pyroxene grains in chondrules are Fe poor (Mg#=93–99). Most of the Fe-rich olivine grains in type IIA chondrules from DaG 978 have low Cr_2_O_3_ contents (<0.2 wt%). A maximum metamorphic temperature of ∼580–680 °C was deduced, based on the compositions of coexisting spinel and olivine^19^. Kamacite and taenite contain 3.8–7.4 wt% and 34.2–44.6 wt% Ni, respectively. Many of the CAIs in DaG 978 contain fine-grained troilite (Supplementary Fig. 1). Lath-shaped olivine grains replacing low-Ca pyroxene occur in some ferromagnesian chondrules and chondrule fragments. Nepheline grains are present as inclusions in some of these lath-shaped olivine grains and as lamellae in anorthite grains. Apatite is common in DaG 978 and has a wide occurrence (described below).

### Petrography and mineralogy of apatite and merrillite

**Figure 1:**
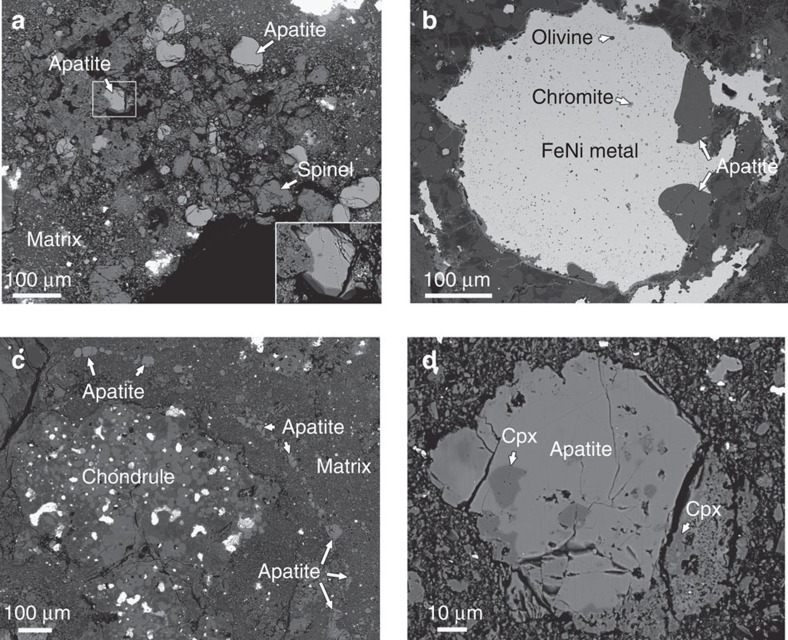
Textural occurrences of apatite in DaG 978. (**a**) Backscattered electron image of apatite grains that are closely associated with a Ca,Al-rich inclusion. The apatite grain size varies from ∼10 to 100 μm. Fine-grained anorthite has been partially altered to nepheline. The outline region contains a subhedral apatite grain with a well-developed crystal form. (**b**) Backscattered electron image of two apatite grains occurring at the margin of a large FeNi metal in a chondrule. The FeNi metal also contains fine-grained inclusions of chromite and olivine. (**c**) Backscattered electron image of apatite grains that resemble a chain of beads in matrix. (**d**) Backscattered electron image of a large apatite grain that is closely associated with high-Ca pyroxene (cpx). The high-Ca pyroxene grains are anhedral to euhedral and vary from 3 to 15 μm in size.

Coarse-grained apatite (>20 μm in size) has three major textural occurrences in DaG 978 (Fig. 1). First, the majority of apatite grains in DaG 978 are closely associated with altered CAIs that can contain secondary minerals such as nepheline, Na-rich plagioclase and troilite (Fig. 1a; Supplementary Fig. 1). Most of the apatite grains associated with altered CAIs are subhedral–euhedral in shape with well-developed crystal forms (Fig. 1a). Second, a few apatite grains occur at the margins of large FeNi metal grains within or outside of chondrules (Fig. 1b). Apatite is rarely included in FeNi metal, which usually contains diopside, olivine, chromite and merrillite as small inclusions. Third, many of the anhedral to euhedral apatite grains occur as isolated crystals in fine-grained matrix of DaG 978. The spatial distribution of some apatite grains in the matrix resembles a chain of beads (Fig. 1c). Regardless of the different textural occurrences, many of the apatite grains in DaG 978 are closely associated with anhedral to euhedral grains of high-Ca pyroxene and/or olivine (Fig. 1d). Some of the high-Ca pyroxene and olivine grains are included in coarse-grained apatite. Merrillite is common in FeNi metal, mainly, as inclusions. Most merrillite grains in DaG 978 are <20 μm in size, only a few grains as large as 50 μm. No merrillite grains are observed within or around altered CAIs in DaG 978. Chemically, electron microprobe results reveal that apatite in DaG 978 is highly Cl rich and has low concentrations of F and calculated H_2_O (Supplementary Table 1). Compared with apatite, merrillite always contains a few weight per cent of MgO, FeO and Na_2_O (Supplementary Table 2). The high-Ca pyroxene closely associated with apatite contains low Al_2_O_3_ and TiO_2_, and high FeO and Na_2_O compared with primary high-Ca pyroxene in CAIs in DaG 978 (Supplementary Table 3, ref. 19).

### REE and oxygen isotopic compositions

**Figure 2:**
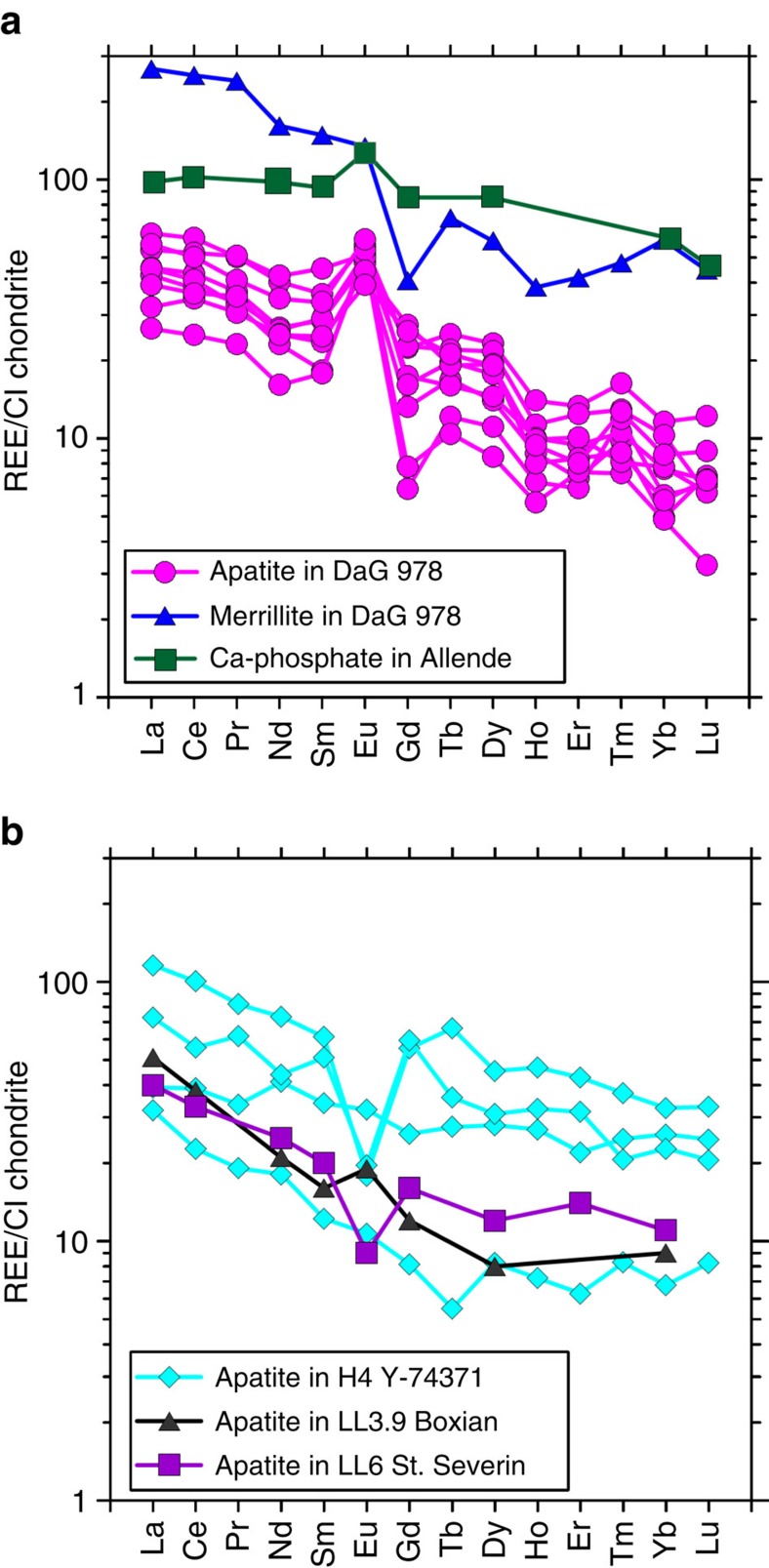
Rare earth element patterns of Ca–phosphate minerals from different chondrites. (**a**) Apatite and merrillite in DaG 978, and the Ca–phosphate in Allende have a positive Eu anomaly with heavy rare earth elements slightly depleted than light rare earth elements. (**b**) The rare earth element compositions in apatite in ordinary chondrites, which were determined by SIMS. Note that apatite grains in less equilibrated ordinary chondrites (H4 Yamato 74371 and LL3.9 Boxian) may contain weak, positive Eu anomalies. Data for Ca–phosphates in ordinary chondrites and Allende are from refs 9, 11, 14.

REEs of nine apatite grains and one merrillite grain from DaG 978 are plotted in Fig. 2, comparing with those of Ca–phosphate minerals from carbonaceous and ordinary chondrites^9^,^11^,^14^. The apatite grains in DaG 978 all have identical REE patterns with positive Eu anomalies (Eu/Eu*=1.5–4.2; Fig. 2). The light REEs are enriched compared with the heavy REEs (La ∼26–62*CI; Lu ∼3–12*CI; Supplementary Table 4). Merrillite contains higher REE concentrations than apatite. However, it also shows a weak, positive Eu anomaly (Fig. 2). Oxygen isotopic compositions of apatite, associated high-Ca pyroxene, olivine in refractory inclusions from DaG 978 and that of bulk DaG 978 (ref. 18) are plotted on a three-isotope oxygen diagram, δ^17^O versus δ^18^O (Supplementary Fig. 2). Oxygen isotopic compositions of both apatite and associated high-Ca pyroxene plot slightly below the terrestrial fractionation line. They have almost identical oxygen isotopic compositions with an average Δ^17^O value of −1.5±3.2‰ (2 s.d.). The oxygen isotopic compositions are similar to those of anorthite and mesostasis in a sapphirine-bearing Al-rich chondrule from DaG 978 (ref. 20), but distinctly different from those of refractory materials in DaG 978 (Supplementary Fig. 2; Supplementary Table 5).

### U–Pb ages of apatite formation

**Figure 3:**
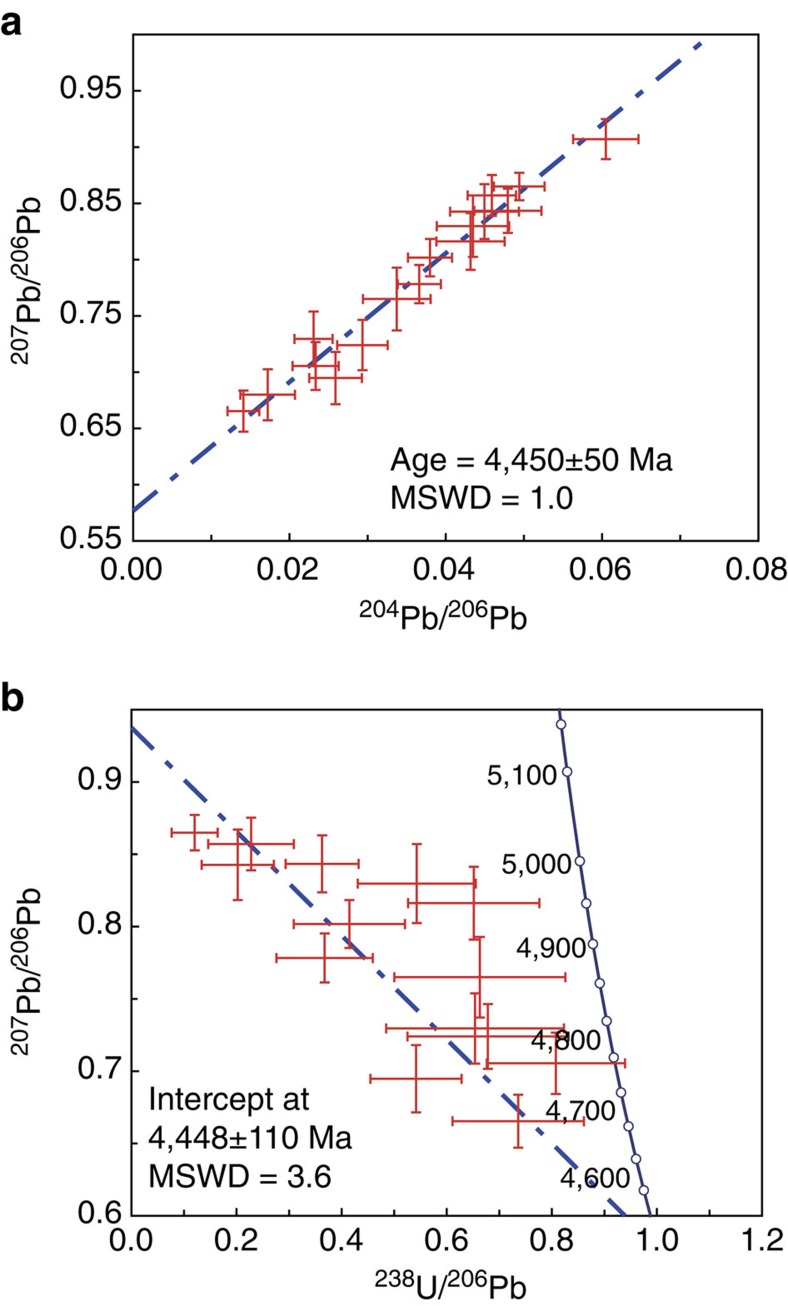
The result of SIMS U–Pb dating of apatite in DaG 978. (**a**) Inverse ^204^Pb/^206^Pb–^207^Pb/^206^Pb isochron diagram. (**b**) The projected diagram onto the ^238^U/^206^Pb–^207^Pb/^206^Pb plane of the total Pb/U isochron in the ^238^U/^206^Pb–^207^Pb/^206^Pb–^204^Pb/^206^Pb three-dimension space. All data suggest that the crystallization age of apatite in DaG 978 is 4,450 Myr. The uncertainties for individual analyses are reported as 2 s.d. The ^207^Pb/^206^Pb isochron and total Pb/U isochron ages were calculated at the 95% confidence level. MSWD, mean square of weighted deviated.

The SIMS measurements on 13 apatite grains reveal that apatite in DaG 978 contains low concentrations of U (0.075–0.178 p.p.m. for most grains) and Th (0.002–0.096 p.p.m. for most grains; Supplementary Table 6). The U/Pb and Pb/Pb isotope ratios calculated by averaging the ratios of measurement counts are plotted on the ^204^Pb/^206^Pb–^207^Pb/^206^Pb diagram and the ^207^Pb/^206^Pb–^238^U/^206^Pb diagram (Fig. 3). The Pb isotope compositions of apatite in DaG 978 define a good ^204^Pb/^206^Pb–^207^Pb/^206^Pb isochron, giving a ^207^Pb/^206^Pb isochron age of 4,450±50 Myr (2 s.d.) (Fig. 3a). The three-dimensional linear regression on the ^207^Pb/^206^Pb–^238^U/^206^Pb diagram gives a total Pb/U isochron age of 4,448±110 Myr (2 s.d.; Fig. 3b). The excellent consistence between the ^207^Pb/^206^Pb isochron age and the total Pb/U isochron age indicates an age of apatite in DaG 978 at ∼4,450 Myr. Meanwhile, the common lead plane intercepts are at ^206^Pb/^204^Pb=15.9±1.5 and ^207^Pb/^204^Pb=14.9±1.3.

## Discussion

The mineralogical features in DaG 978 indicate that both metamorphic process and metasomatic process have affected this chondrite^19^. The main evidence of metamorphism in DaG 978 is the chemical changes of some primary minerals (that is, olivine, chromite and FeNi metal) in chondrules and refractory inclusions. The chemical changes of primary minerals require thermally driven volume diffusion of some elements (that is, Mg, Fe, Cr and Ni). The olivine–chromite thermometer suggests a maximum equilibrium temperature of ∼580–680 °C, indicating mild thermal metamorphism^19^. The real metamorphic temperature could be lower than this temperature, because the olivine–chromite pairs in a type 3.5 chondrite might not have reached chemical equilibrium^21^. On the other hand, lath-shaped olivine, nepheline, Ca–phosphates and troilite in CAIs could not have formed with the metamorphic process alone. Instead, they could have formed during metamorphism in the presence of fluid (that is, replacement of primary minerals by fluids and/or direct precipitation from fluids^19^,^22^). For example, the formation of apatite that is closely associated with CAIs requires that P and Cl migrated from a source outside the CAIs. Without the presence of fluid, it is difficult to interpret the migration of these elements in DaG 978 (ref. 11). Lath-shaped olivine is widely interpreted as a product of fluid-assisted metamorphism^6^,^22^. The lack of phyllosilicate minerals in DaG 978 indicates that the fluid–mineral interaction should have taken place at temperatures of >200 °C (refs 4, 6). This requirement is generally consistent with the temperature deduced from the olivine–chromite geothermometry and those (∼400 °C or higher) for other chondrites of around type 3.5 (refs 6, 23). Therefore, the thermal event drove the diffusion of some elements in primary minerals. It might also result in the onset of fluid activity in DaG 978. During or intermediately subsequent to the thermal event, the fluids interacted with the minerals in DaG 978 and formed the secondary minerals. In summary, the apatite in DaG 978 should be a product of fluid-assisted metamorphism on the parent body^11^.

Close textural association between apatite, and both FeNi metal and altered CAIs indicates that FeNi metal and CAIs could be the main sources of P and Ca for the formation of apatite in DaG 978, respectively. Chondrule mesostasis could be another source of P because some mesostasis in primitive chondrites contain P_2_O_5_ up to 3.5 wt% (ref. 24). The source of Cl-rich fluid cannot be constrained based on the current observation. However, the partition coefficients of F and OH between apatite and aqueous fluids are much higher than that of Cl (ref. 25). Therefore, the low contents of F and calculated H_2_O in apatite from DaG 978 indicate that the fluids from which apatite formed must be highly depleted in F and H_2_O and highly enriched in Cl.

Although both the apatite in DaG 978 and those in metamorphosed ordinary chondrites (OCs) could have formed though fluid-assisted metamorphism, their REE geochemistry and petrographic textures may reflect various contributions from metamorphic processes and metasomatic processes. Most of apatite grains in type 4–6 OCs exhibit negative Eu anomalies^11^,^26^. The negative Eu anomaly of the apatite from type 4–6 OCs could be a result of Eu equilibrium between apatite and recrystallized plagioclase during thermal metamorphism, with most Eu incorporated into plagioclase^9^,^11^. However, the apatite in DaG 978 has a positive Eu anomaly, similar to those observed in the Allende meteorite (type 3) and some of the apatite grains in type 3.9 and type 4 OCs^9^,^11^,^14^. Such positive Eu anomalies indicate the lack of chemical equilibrium between apatite and plagioclase. Instead, they may reflect the chemical features of fluids from which apatite formed^9^. The petrographic textures of apatite in DaG 978 do support that fluid–mineral interaction should have played a key role for its formation. For example, fluid activity can well interpret the euhedral apatite crystals in matrix and those associated with CAIs. On the contrast, the apatite grains in highly metamorphosed OCs are mainly irregular in shape^11^. In addition, a pathway of fluid migration can best interpret the chain-like distribution of some apatite grains in matrix (Fig. 1c). In summary, the geochemical and petrographic features of apatite in DaG 978 also reflect the onset of mild metamorphic process in the presence of an aqueous fluid in a type 3 chondrite parent body. Thus, the age of apatite records the timing of fluid activity in DaG 978.

The ^207^Pb/^206^Pb isochron age (4,450±50 Myr) of the apatite obtained in this study has two important implications. First, in the literature, all ages (short-lived isotope chronologies, Sr isotope composition and Rb–Sr isotope chronology) about fluid activity in ESS are relative ages or model ages^7^,^27^,^28^. The apatite age obtained in this study is the first absolute age of fluid activity in ESS up to date. Second, the age of apatite in DaG 978 is ∼117 Myr younger than the oldest CAI^29^. This indicates that fluid activity may extend for a long time interval in ESS, from a few Myr to ∼117 Myr after CAI formation. It has been suggested that the solar nebula has a lifetime of ∼10 Myr (ref. 30). Therefore, the young age of the apatite in DaG 978 demonstrates that this fluid activity must have taken place on the parent body^7^.

Decay of short-lived radionuclides and collisional heating has been proposed as significant heat sources to thermal metamorphism of OCs^21^. For example, the main heating source for fluid activity within ∼15 Myr after CAI formation could be the decay of short-lived radionuclides (for example, ^26^Al and ^60^Fe (refs 7, 31)). However, short-lived radionuclides cannot be a viable heat source for a thermal event that occurred 117 Myr after CAI formation. Instead, collisional heating in late ESS may provide enough heat to activate the metamorphic event in the presence of fluid on the parent body of DaG 978. It has been suggested that there are frequent impact events at 4.47±0.03 Ga in the main belt after the Moon-forming impact event, based on Ar–Ar ages of ordinary chondrites^32^ and phosphate U–Pb ages of ordinary chondrites^16^,^17^, and dynamic modelling^33^. On the basis of this excellent consistency between the apatite ^207^Pb/^206^Pb isochron age and the timing of impact events proposed in the literature, it is most likely that an impact event may have provided the heat to activate the metamorphic event at 4,450 Myr on the parent body of DaG 978.

In addition, recent studies suggested that some large asteroids (that is, Ceres) have water evaporation from localized regions, indicating subsurface fluid activity^34^. Some of these water evaporation events could be related to impact events. Some of the evaporation events could be due to cryo-volcanism^34^. Thus, we cannot totally exclude the possibility that DaG 978 might have derived from the subsurface of a large asteroid, where fluid activity might have lasted up to 117 Myr after CAI formation.

In summary, the apatite in DaG 978 has an origin by fluid-assisted metamorphism and was formed at 4,450 Myr, ∼117 Myr later than CAI formation. Its age is the first absolute age about fluid activity in ESS. The late formation of apatite in DaG 978 represents a young metamorphic event in the presence of fluid in the parent body setting. An impact event at ∼4,450 Myr may have provided the heat to activate this fluid activity in late ESS. Alternatively, this meteorite might be ejected from a large (possibly Ceres like) asteroid, where subsurface fluid activities could last up to ∼117 Myr after CAI formation.

## Methods

### Petrography and major element analysis

Petrographic textures of apatite in DaG 978 were mainly observed with JEOL 7000F field-emission scanning electron microscope (SEM) at Hokkaido University and JEOL 6490 SEM at Nanjing University. Both SEM instruments are operated at an accelerating voltage of 15 kV. Mineral chemistry of apatite was determined using a JEOL 8100 electron probe micro-analyzer (EPMA) at Nanjing University. A defocused beam (10 μm in diameter) at a beam current of 10 nA and an accelerating voltage of 15 kV was used. A few natural and synthetic materials were used standards. All EPMA data are reduced with the ZAF (atomic number–absorption–fluorescence) correction procedure. Before EPMA analysis, all Ca–phosphate minerals were qualitatively measured by energy dispersive spectrometers (EDS) installed on the SEM instruments. Since SEM–EDS results show high chlorine and a very low intensity of fluorine, Cl and F are the first and second elements to be measured, respectively.

### Oxygen isotope systematics

Oxygen isotope compositions of apatite and associated silicate minerals were determined with the Cameca IMS-1270 instrument at Hokkaido University. Before measurements, the polished sections of DaG 978 were carbon-coated. The primary ion beam was mass filtered positive Cs^+^ ions of 20 keV and the beam spot size was 8–10 μm in diameter. A primary beam current of 100 pA was used to obtain a count rate of negative ^16^O ions of ∼2 × 10^7^ c.p.s. A normal-incident electron gun was used for charge compensation of the sputtered area. A mass resolving power of ∼5,500 was used to separate ^17^O from ^16^OH. Negative ^16^O ions were detected with an axial Faraday cup, while negative ^17^O and ^18^O ions were detected with an axial electron multiplier detector, in magnetic peak-jumping mode. The instrumental mass fractionation effect was corrected using San Carlos olivine. The reported uncertainties of the individual analyses are expressed in 2 s.d., which were estimated by considering both the internal error of each measurement and the reproducibility of the standard measurements. After the measurements, all spots were evaluated using filed-emission SEM.

### REE geochemistry

The REE compositions of apatite and merrillite were determined with the Cameca IMS-6f instrument at Hokkaido University. Before measurements, the polished sections of DaG 978 were carbon-coated. The procedure was similar to that described in refs 35, 36. The primary beam was mass filtered ^16^O^−^ of –14.5 keV and irradiated on the sample surface to a diameter of ∼25 μm. The primary beam current is 10–15 nA. Kinetic energy filtering was used to reduce interferences from molecular ions by offsetting the sample acceleration voltage (–100 eV). The energy bandwidth was 20 eV. The exit slit was set to a mass resolving power of ∼500. Positive secondary ion intensities were counted for 10 s with an electron multiplier detector in magnetic peak-jumping mode. Each analysis included 15 cycles. Relative sensitivity factors between secondary ion intensity and concentration for each REE (relative to Ca) were determined using the Takashima augite, for which REE contents have been well determined by instrumental neutron activation analysis^37^.

### Uranium–lead isotopic systematics

The U–Pb isotopic system of apatite was determined using the Cameca IMS-1280HR instrument at the Institute of Geology and Geophysics, Chinese Academy of Sciences, China, following the analytical procedure of refs 15, 38. Before measurements, the polished sections of DaG 978 were carbon-coated. The O_2_^−^ primary beam was accelerated at −13 kV, with an intensity of ∼10 nA. The ellipsoidal spot is ∼20 × 30 μm in size. The secondary ions were extracted at an initial energy of 10 keV. The entrance slit and field aperture were set to a mass resolution power of ∼8,000. The energy slit was closed to a bandwidth of 60 eV to reduce the energy dispersion. Detection of secondary ions took the advantage of both mono-collector mode and multi-collector mode. For each analysis in this study, secondary ion beam intensities of ^40^Ca_2_^31^P^16^O_4_^+^, ^238^U^+^ and ^238^U^16^O_2_^+^ were detected in L1-EM by peak-jumping sequences. In the second sequence, ^204^Pb, ^206^Pb and ^207^Pb were detected with multi-collector electron multipliers L2, L1 and C, respectively. In the third sequence, ^238^U, ^232^Th^16^O and ^238^U^16^O were measured with L1, H1 and H2, respectively. The Pb/U ratios were calibrated with an empirical correlation between Pb^+^/U^+^ and UO^2+^/U^+^ ratios, normalized to the 1,160 Myr apatite standard from the Prairie Lake alkaline–carbonatite complex in Ontario, Canada^13^,^15^. Each analysis included 10–20 cycles. The U/Pb and Pb/Pb isotope ratios were calculated by averaging the ratios of measurement counts. Uncertainties for individual analyses are reported as 2 s.d. The ^207^Pb/^206^Pb isochron and total Pb/U isochron ages were calculated at the 95% confidence level using the Isoplot/Ex 3.0 software^39^.

### Data availability

The data shown and discussed in this paper are presented in the Supplementary Information.

## Additional information

**How to cite this article**: Zhang, A-C. *et al*. Young asteroidal fluid activity revealed by absolute age from apatite in carbonaceous chondrite. *Nat. Commun.* 7:12844 doi: 10.1038/ncomms12844 (2016).

## Supplementary Material

Supplementary Information Supplementary figure 1-2, Supplementary table 1-6
