# Supplementary material for: Young asteroidal fluid activity revealed by absolute age from apatite in carbonaceous chondrite
Source: Nat Commun. 2016 Sep 29;7:12844. doi: 10.1038/ncomms12844 (PMC5056421; doi:10.1038/ncomms12844)
Supplement: Supplementary Information — Supplementary figure 1-2, Supplementary table 1-6 [file ncomms12844-s1.pdf]

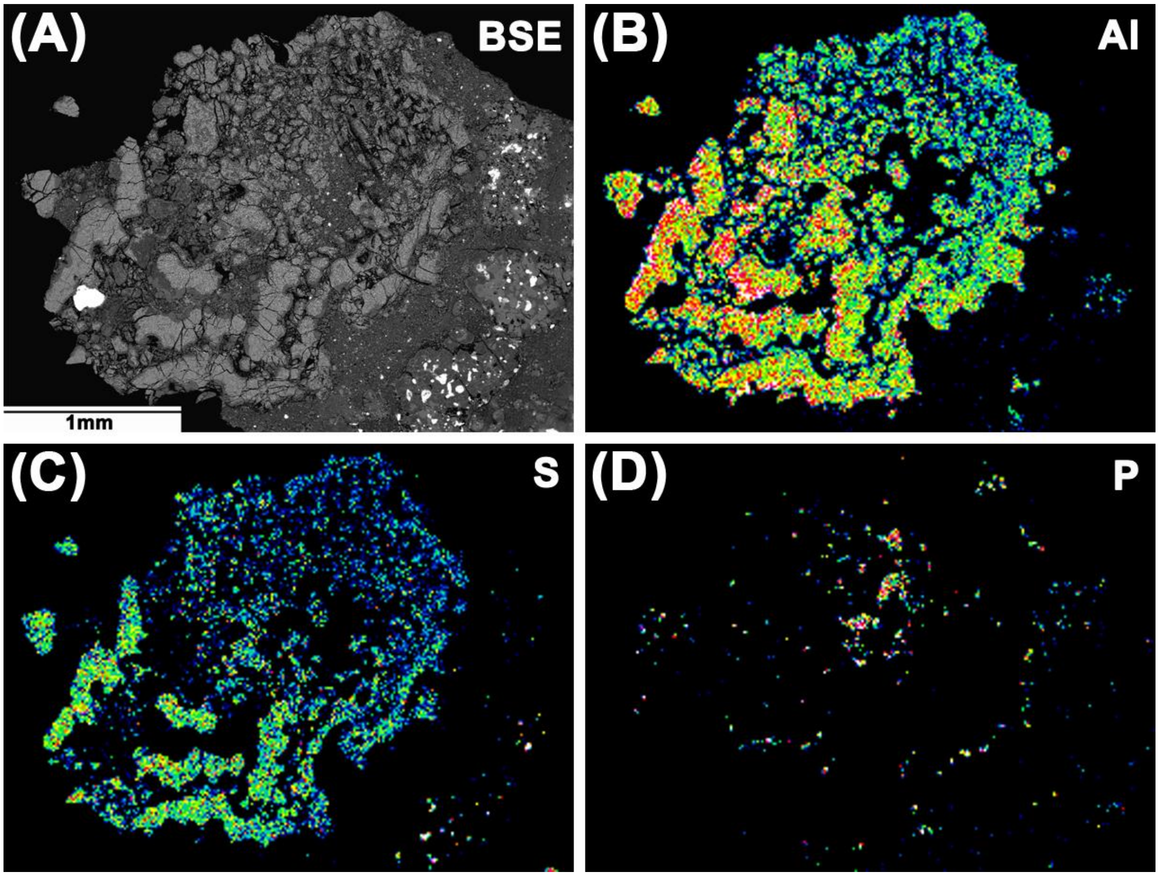

Supplementary Figure 1 | Backscattered electron image (a) and X-ray elemental mapping results (b-d) of a sulfurized CAI and closely associated apatite grains. Note that P (representing apatite) is highly enriched around the Al-rich regions (representing Ca,Al-rich inclusion).

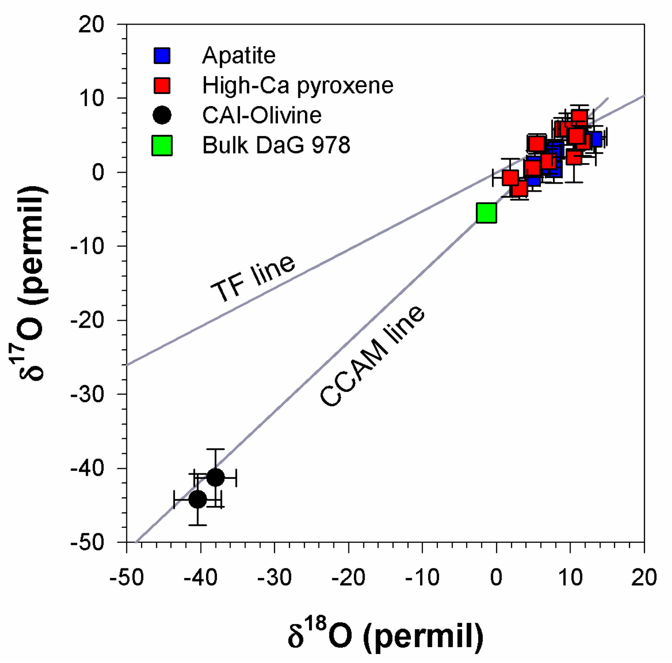

Supplementary Figure 2 | Oxygen isotope compositions of apatite and its associated high-Ca pyroxene in DaG 978. Oxygen isotope compositions of CAI olivine from DaG 978 and that of the bulk DaG 978 (Supplementary Ref 1) are also plot for comparison. The error bars are 2 s.d.

Supplementary Table 1 | EPMA compositions (wt%) of apatite in DaG 978

	1	2	3	4	5	6
P_2O_5	39.95	40.77	39.68	40.73	40.26	40.74
FeO	0.24	0.43	0.38	0.29	0.77	0.31
MnO	<0.02	0.04	<0.02	0.02	0.05	<0.02
CaO	53.17	53.41	53.93	53.38	53.46	53.45
Na_2O	0.41	0.43	0.38	0.32	0.31	0.44
F	0.22	0.07	0.10	0.16	0.10	0.19
Cl	5.39	5.25	5.61	5.42	5.20	5.01
H_2O*	0.22	0.36	0.21	0.27	0.34	0.36
Total	99.60	100.8	100.3	100.6	100.5	100.5

*Concentrations of H_2O were calculated based on the assumption of F+Cl+OH=1 in apatite crystal structure.

Supplementary Table 2 | EPMA compositions of merrillite in DaG 978

Element	1	2	3	4	5	6	7	8	10
P_2O_5	45.71	45.02	45.48	45.19	45.22	43.22	45.87	45.65	44.66
SiO_2	0.04	0.02	0.04	0.09	0.07	0.16	0.06	0.08	0.16
MgO	3.65	3.65	3.71	3.72	3.69	3.59	3.69	3.66	3.66
FeO	1.65	1.88	1.78	2.91	2.59	4.07	2.13	2.11	2.57
MnO	0.02	0.04	0.03	<0.02	<0.02	<0.02	0.02	<0.02	<0.02
CaO	45.86	45.91	45.81	45.31	45.60	44.61	45.64	45.75	45.39
Na_2O	2.81	2.80	2.82	2.80	2.74	2.69	2.80	2.84	2.83
K_2O	0.03	<0.02	0.02	<0.02	0.02	0.02	0.03	0.03	0.03
Total	99.77	99.32	99.69	100.0	99.93	98.36	100.2	100.1	99.30

Supplementary Table 3 | EPMA compositions of high-Ca pyroxene associated with apatite compared with those in refractory inclusions

	High-Ca pyroxene associated with apatite					High-Ca pyroxene in refractory inclusions				
SiO_2	52.27	53.16	53.12	53.47	52.29	53.39	40.29	50.13	54.21	40.17
TiO_2	<0.02	<0.02	<0.02	<0.02	<0.02	0.05	4.95	0.45	0.20	5.43
Al_2O_3	0.32	0.10	0.07	0.13	0.81	2.98	17.69	7.22	2.16	18.58
Cr_2O_3	0.04	0.22	0.19	0.36	0.11	0.05	0.08	0.07	0.03	0.05
MgO	12.03	14.40	13.98	15.10	12.55	16.54	9.83	14.58	17.79	9.98
FeO	9.46	5.74	7.92	5.85	7.93	0.49	0.60	0.53	0.40	0.55
MnO	0.04	0.10	0.08	<0.02	0.07	<0.02	0.06	<0.02	<0.02	<0.02
CaO	24.44	25.19	24.95	24.88	24.63	25.77	24.78	25.46	24.87	25.11
Na_2O	0.07	0.12	0.05	0.12	0.11	<0.02	0.03	0.04	<0.02	<0.02
Total	98.67	99.03	100.4	99.91	98.50	99.27	98.31	98.48	99.66	99.87

Supplementary Table 4 | REE concentrations (ppm) in apatite and merrillite from DaG 978

	a-1	σ	a-2	σ	a-3	σ	a-5	σ	a-6	σ	a-7	σ	a-8	σ	a-10	σ	a-11	σ	Mrl-1	σ
La	14.5	0.3	12.6	0.2	10.7	0.3	10.0	0.2	10.6	0.3	6.2	0.2	13.2	0.3	7.5	0.2	9.2	0.2	62.9	0.7
Ce	35.8	0.5	31.4	0.4	26.2	0.4	22.9	0.5	24.7	0.4	15.1	0.3	30.3	0.5	20.9	0.4	21.9	0.4	152.6	1.2
Pr	4.5	0.1	4.5	0.2	3.3	0.1	3.0	0.1	2.9	0.2	2.1	0.1	3.7	0.2	2.7	0.1	3.2	0.1	21.5	0.5
Nd	18.2	0.6	19.2	0.6	12.1	0.6	11.9	0.5	10.4	0.5	7.3	0.4	15.7	0.8	11.4	0.6	11.4	0.7	72.9	1.4
Sm	5.3	0.2	6.7	0.4	4,2	0.2	4.3	0.4	2.7	0.4	2.6	0.3	4.9	0.5	3.5	0.4	3.6	0.3	21.9	1.0
Eu	3.1	0.3	2.9	0.1	2.7	0.2	2.9	0.2	2.8	0.2	3.0	0.3	3.3	0.1	2.5	0.3	2.2	0.3	7.5	0.3
Gd	4.4	0.3	4.5	0.4	2.6	0.2	3.4	0.3	1.3	0.2	1.5	0.2	3.2	0.4	5.4	0.3	5.1	0.3	8.0	0.5
Tb	0.9	0.1	0.8	0.1	0.6	0.1	0.6	0.1	0.4	0.1	0.4	0.1	0.7	0.1	0.7	0.1	0.8	0.1	2.6	0.1
Dy	5.6	0.4	5.3	0.2	3.4	0.3	3.5	0.2	2.7	0.2	2.1	0.3	4.3	0.4	4.7	0.5	4.6	0.4	14.0	0.7
Ho	0.8	0.1	0.6	0.1	0.5	0.1	0.6	0.1	0.4	0.1	0.3	0.1	0.6	0.1	0.4	0.1	0.5	0.1	2.1	0.1
Er	2.1	0.2	2.0	0.3	1.1	0.2	1.5	0.2	1.0	0.2	1.2	0.1	1.6	0.2	1.3	0.2	1.3	0.2	6.7	0.5
Tm	0.4	0.1	0.3	0.1	0.2	0.1	0.3	0.1	0.3	0.1	0.2	0.1	0.2	0.1	0.2	0.1	0.3	0.1	1.2	0.1
Yb	1.9	0.1	1.7	0.3	0.8	0.1	1.2	0.2	1.0	0.2	0.8	0.1	1.3	0.1	0.9	0.2	1.4	0.2	9.5	0.5
Lu	0.3	0.1	0.1	0.1	0.2	0.1	0.2	0.1	0.2	0.1	0.1	0.1	0.2	0.1	0.2	0.1	0.2	0.1	1.1	0.1

Supplementary Table 5 | Oxygen isotope compositions (‰) of apatite and the closely associated high-Ca pyroxene in DaG 978

	Mineral	$\delta^{17}O$	2σ	$\delta^{18}O$	2σ	$\Delta^{17}O$
dag@6	apatite	0.4	1.9	7.7	1.1	-3.6
dag@7	apatite	0.7	2.0	6.2	1.0	-2.5
dag@13	apatite	-0.7	1.7	4.8	1.0	-3.3
dag@20	apatite	4.8	3.7	11.3	3.5	-1.1
dag@21	apatite	1.1	1.8	5.1	1.1	-1.5
dag@23	apatite	3.1	1.2	8.0	0.9	-1.1
dag@29	apatite	4.5	1.8	13.1	1.4	-2.4
dag@30	apatite	2.5	1.8	7.6	1.1	-1.4
dag@8	pyroxene	-2.2	1.5	3.1	0.9	-3.8
dag@9	pyroxene	0.6	1.0	4.8	1.1	-1.9
dag@10	pyroxene	1.5	1.7	7.0	1.0	-2.2
dag@11	pyroxene	5.9	1.5	8.9	1.4	1.2
dag@19	pyroxene	2.1	3.4	10.4	3.0	-3.3
dag@22	pyroxene	-0.7	2.6	1.8	2.3	-1.7
dag@24	pyroxene	3.9	1.3	5.4	1.3	1.0
dag@27	pyroxene	5.9	2.1	9.6	1.3	0.9
dag@28	pyroxene	7.3	1.7	11.2	1.9	1.5
dag@31	pyroxene	4.1	1.9	11.6	1.3	-1.9
dag@32	pyroxene	4.9	2.0	10.7	1.3	-0.7
dag@15	CAI-olivine	-44.2	3.5	-40.5	3.2	-23.2
dag@16	CAI-olivine	-41.3	3.9	-38.0	2.8	-21.5

Supplementary Table 6 | SIMS U-Pb isotopic data of apatite from DaG 978

Sample/ spot #	$^{238}U/^{206}Pb$	±σ %	$^{207}Pb/^{206}Pb$	±σ %	$^{204}Pb/^{206}Pb$	±σ %	[U] ppm	[Th] ppm	Th/U meas
D01@1	0.663	12.3	0.765	1.82	0.034	6.42	0.146	0.003	0.023
D02@1	0.678	11.3	0.724	1.54	0.029	5.51	0.124	0.012	0.097
D03@1	0.651	9.6	0.816	1.54	0.043	5.06	0.101	0.005	0.049
D03@2	0.543	10.3	0.830	1.65	0.043	5.33	0.075	0.002	0.031
D04@1	0.363	9.6	0.843	1.17	0.048	4.48	0.123	0.043	0.349
D05@1	0.808	8.1	0.705	1.50	0.023	6.34	0.140	0.056	0.398
D05@2	0.736	8.5	0.665	1.38	0.014	7.20	0.172	0.096	0.557
D05@3			0.680	1.67	0.017	10.14			
D06@1	0.654	12.9	0.730	1.66	0.023	5.27	0.178	0.033	0.187
D07@1	0.542	8.0	0.695	1.67	0.026	6.50	0.088	0.013	0.145
D08@1	0.120	18.2	0.865	0.71	0.049	3.29	0.138	0.011	0.082
D09@1			0.907	0.98	0.060	3.46			
D10@1	0.415	12.7	0.802	1.04	0.038	3.70	0.145	0.006	0.038
D11@1	0.368	12.5	0.778	1.09	0.037	3.78	0.108	0.011	0.104
D12@1	0.202	17.0	0.843	1.45	0.045	4.89	0.091	0.039	0.431
D13@1	0.228	17.9	0.857	1.06	0.046	3.37	0.103	0.008	0.077

Supplementary References

1. Choe, W. H, Huber, H., Rubin, A. E., Kallemeynm G. W. & Wasson, J. T. Compositions and taxonomy of 15 unusual carbonaceous chondrites. *Meteoritics & Planetary Science* **45**, 531–554 (2010).
